# Anaerobic digestion of process water from hydrothermal treatment processes: a review of inhibitors and detoxification approaches

**DOI:** 10.1186/s40643-024-00756-6

**Published:** 2024-05-07

**Authors:** Mei Zhou, Kayode Taiwo, Han Wang, Jean-Nepomuscene Ntihuga, Largus T. Angenent, Joseph G. Usack

**Affiliations:** 1 Environmental Biotechnology Group, Department of Geosciences, University of Tübingen, Schnarrenbergstr. 94-96, 72076 Tübingen, Germany; 2 Department of Food Science and Technology, University of Georgia, 100 Cedar Street, Athens, GA 30602 USA; 3 Max Planck Institute for Biology Tübingen, AG Angenent, Max Planck Ring 5, 72076 Tübingen, Germany; 4 Department of Biological and Chemical Engineering, Aarhus University, Gustav Wieds vej 10D, 8000 Aarhus C, Denmark; 5 The Novo Nordisk Foundation CO2 Research Center (CORC), Aarhus University, Gustav Wieds vej 10C, 8000 Aarhus C, Denmark; 6 Cluster of Excellence, Controlling Microbes to Fight Infections, University of Tübingen, Auf der Morgenstelle 28, 72074 Tübingen, Germany; 7 New Materials Institute, University of Georgia, 220 Riverbend Rd, Athens, GA 30602 USA; 8 Institute for Integrative Agriculture, Office of Research, University of Georgia, 130 Coverdell Center, 500 D.W. Brooks Dr., Athens, GA 30602 USA

**Keywords:** Hydrothermal treatment, Process water, Anaerobic digestion, Biogas, Inhibition, Detoxification

## Abstract

Integrating hydrothermal treatment processes and anaerobic digestion (AD) is promising for maximizing resource recovery from biomass and organic waste. The process water generated during hydrothermal treatment contains high concentrations of organic matter, which can be converted into biogas using AD. However, process water also contains various compounds that inhibit the AD process. Fingerprinting these inhibitors and identifying suitable mitigation strategies and detoxification methods is necessary to optimize the integration of these two technologies. By examining the existing literature, we were able to: (1) compare the methane yields and organics removal efficiency during AD of various hydrothermal treatment process water; (2) catalog the main AD inhibitors found in hydrothermal treatment process water; (3) identify recalcitrant components limiting AD performance; and (4) evaluate approaches to detoxify specific inhibitors and degrade recalcitrant components. Common inhibitors in process water are organic acids (at high concentrations), total ammonia nitrogen (TAN), oxygenated organics, and N-heterocyclic compounds. Feedstock composition is the primary determinant of organic acid and TAN formation (carbohydrates-rich and protein-rich feedstocks, respectively). In contrast, processing conditions (e.g., temperature, pressure, reaction duration) influence the formation extent of oxygenated organics and N-heterocyclic compounds. Struvite precipitation and zeolite adsorption are the most widely used approaches to eliminate TAN inhibition. In contrast, powdered and granular activated carbon and ozonation are the preferred methods to remove toxic substances before AD treatment. Currently, ozonation is the most effective approach to reduce the toxicity and recalcitrance of N and O-heterocyclic compounds during AD. Microaeration methods, which disrupt the AD microbiome less than ozone, might be more practical for nitrifying TAN and degrading recalcitrant compounds, but further research in this area is necessary.

**Graphical Abstract:**

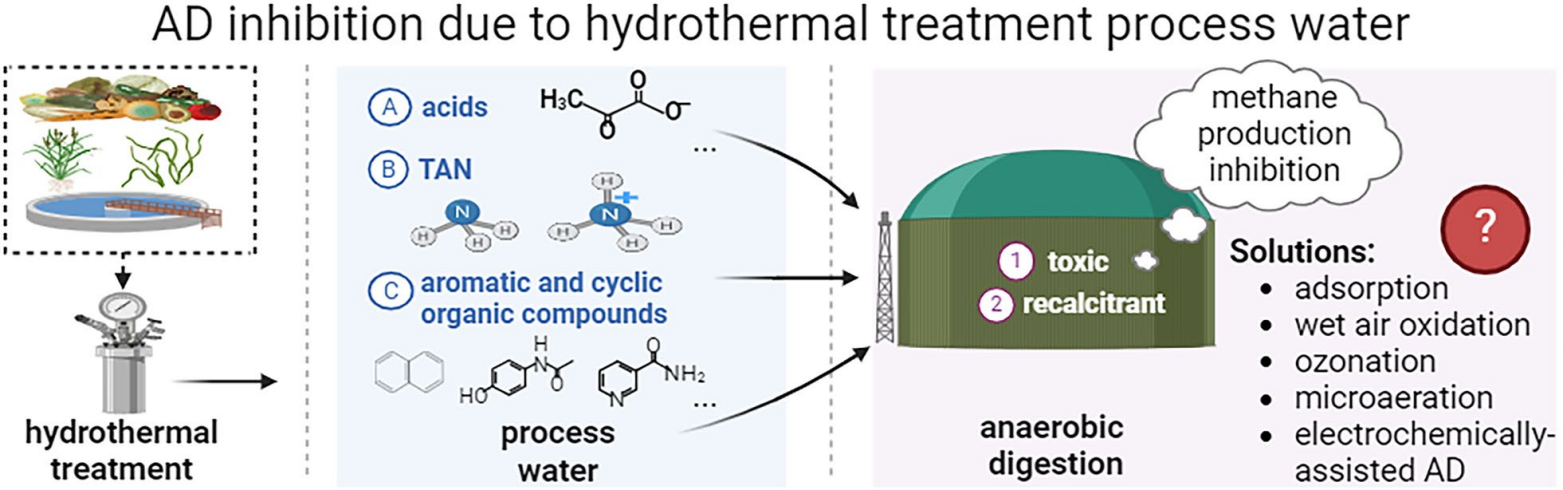

## Introduction

Anaerobic digestion (AD) is a biological process by which microbes convert complex and simple organic substrates into mainly methane and carbon dioxide (Angenent et al. 2004). AD plays a considerable role within the bioeconomy due to its ability to recover energy from various organic waste streams. Concurrently, it mineralizes nutrients, yielding a more effective fertilizer as digestate (Pecchi and Baratieri 2019). However, the hydrolysis of particulate substrates is often the rate-limiting step during AD. Low hydrolysis rates also cause lower biogas yields due to the incomplete conversion of the particulate substrates (Cabrera et al. 2023).

Alongside traditional biological treatment, hydrothermal treatment–a physicochemical process analogous to pyrolysis–is emerging for resource recovery from less biodegradable biomass. Compared to pyrolysis, however, hydrothermal processing is particularly advantageous for treating high-moisture biomass because the reaction medium is water, rendering biomass drying unnecessary (Tekin et al. 2014). Also, the hydrothermal reaction is intense. The reaction applies high temperatures (250–374 ℃) and pressures (4–22 MPa) for short durations (10–200 min). These reaction conditions enable faster throughputs of particulate materials compared to biological treatments while simultaneously pasteurizing the material (Ruiz et al. 2013; Tekin et al. 2014). The process generates several products, including hydrochar, bio-crude oil, and gaseous products. Product specificity can be controlled to a certain extent by changing process variables, such as the reaction temperature, pressure, and time, and by adding specific catalysts (Adams et al. 2018).

However, because water is used as the reaction medium and hydrothermal processing is suitable for feedstocks with high moisture content (i.e., > 65% water), the process generates considerable amounts of high-organic strength wastewater alongside the non-aqueous products (i.e., hydrochar, biocrude oil) (Kambo et al. 2018). This wastewater–also called the aqueous phase byproduct–contains 3.8% to 55% of the initial carbon in the raw biomass. For instance, hydrothermal process water retained > 30% of the carbon that was initially present in the lignocellulosic biomass, food waste, and certain algae (Leng et al. 2020). Moreover, process water cannot be directly discharged into the environment because of its high organic load, which also contains phenols, nitrogenated compounds, and other potentially toxic substances (Elliott et al. 2015; Usman et al. 2022; Yu et al. 2011).

Previous work has demonstrated the benefits of coupling hydrothermal treatment with AD treatment of process water (Cabrera et al. 2023; Romero et al. 2023). During AD, the dissolved organic carbon in the process water is converted into biogas (mainly CH_4_ and CO_2_). Marin-Batista et al. (2020) reported that overall energy, including combustion of hydrochar and biogas from AD treatment of process water, increased more than three times compared to sole AD treatment of dairy manure. Combining hydrothermal and AD also yielded a higher energetic return than hydrothermal treatment alone (Posmanik et al. 2017). Therefore, when coupled, the overall sustainability of the two technologies is improved, leading to more environmental impact reductions and economic benefits.

However, the varied and unpredictable composition of process water makes devising reliable AD treatment strategies difficult. Process water often contains high proportions of short-chain organic acids (*e.g.,* acetic acid), oxygenates (*e.g.,* alcohols, ketones, phenols, and cyclic oxygenates), N-containing compounds (*e.g.,* amino acids, amines, N-heterocyclic compounds, and their derivatives), and inorganic materials (*e.g.,* ammonium, phosphorous, and various metals) (Cabrera and Labatut 2021; Leng and Zhou 2018). Some of these organic compounds are amenable to AD treatment, while others can be inhibitory depending on their concentration (Posmanik et al. 2017). Inhibition of the AD process will also depend on other factors, including the level of biomass acclimation, exposure time, feeding regime, substrate composition, and operating conditions (*e.g.,* pH and temperatures) (Chen et al. 2008; Zhou et al. 2015).

AD operators could conduct toxicology assays to characterize the risk of AD inhibition from specific process waters (Zhou et al. 2015). However, this approach is impractical, given the compositional variation of process water, the variety of toxic compounds present, and the unique characteristics of any given AD microbiome. Instead, we conducted a systematic review and critical analysis of existing literature to provide practical, over-arching insights regarding key indicators, process tolerances/limits, underlying inhibitory mechanisms, and potential detoxification strategies of process water from a biological perspective. This review will serve as a practical reference for scientists and practitioners intending to understand, apply, and optimize the digestibility of process water resulting from hydrothermal processes.

## Anaerobic biodegradability of process water

### Effects of feedstocks in hydrothermal treatment

Feedstocks for hydrothermal treatment contain varying proportions of macromolecules such as proteins, lipids, lignin, carbohydrates, cellulose, and hemicellulose (Table 1). For instance, agricultural residues mainly comprise carbohydrates, lignin, cellulose, and hemicellulose (Li and Cai 2022; Seyedsadr et al. 2018; Tian et al. 2020). Protein and lipids concentrations are higher in manure, sewage, algal biomass, and food waste feedstocks (Akarsu et al. 2019; Bayat et al. 2021; Biller and Ross 2011; Chen et al. 2014; Cheng et al. 2020; Gupta et al. 2020; Liu et al. 2022, 2019; Lu et al. 2018; Motavaf and Savage 2021; Wang et al. 2017). The hydrothermal process hydrolyzes the macromolecules in the feedstock into smaller molecules, including sugars, amino acids, and fatty acid monomers, which serve as substrates for acidogenesis, acetogenesis, and methanogenesis during AD treatment (Basar et al. 2023). Thus, hydrothermal treatment effectively increases AD throughput and yields by circumventing biological hydrolysis, which is often the rate-limiting step (Angenent et al. 2022).

**Table 1:** Proximate analysis of chemical composition in hydrothermal treatment feedstocks

	Agricultural Residues	Algal biomass	Manure	Food Waste
Lipid (%)	–	5–32	10.6	15.7–21.9
Protein (%)	–	36.4–65	26.4	17.8–27.5
Hemicellulose (%)	3.67–31.09	3.5	15.0–34.0	27.4
Cellulose (%)	16.88–46.33	14.4	12.2–28.77	3.4
Lignin (%)	7.0–19.82	5.7	5.4–29.45	4.2
Ash (%)	4.43–15.10	8.9–47.5	10.0–18.30	1.1–8.1
Carbohydrate (%)	–	8–40	–	52.8–58.9

The data were collected from multiple studies: Agricultural Residues (Li and Cai 2022; Seyedsadr et al. 2018; Tian et al. 2020); Algal biomass (Biller and Ross 2011; Chen et al. 2014; Wang et al. 2017); Manure (Liu et al. 2022, 2019; Lu et al. 2018); Food Waste (Akarsu et al. 2019; Bayat et al. 2021; Cheng et al. 2020; Gupta et al. 2020; Motavaf and Savage 2021)

The composition of feedstocks in hydrothermal treatment plays a vital role in the characteristics, and thus the biodegradability of process water (Posmanik et al. 2017). During hydrothermal treatment, multiple reactions occur simultaneously and sequentially during the reaction period. The interaction of multiple factors, including reactant composition and processing conditions, dictates the type and progression of reactions. For example, the treatment of carbohydrates and proteins at moderately high temperatures (i.e., 250–350 °C) favors Maillard reactions, which leads to greater formation of phenolic and N-heterocyclic compounds compared to the treatment of carbohydrates and lipids at lower temperatures (i.e., < 250 °C) (Basar et al. 2023). The various water-soluble reaction products that form during hydrothermal treatment will then fractionate in the aqueous phase, ultimately affecting the biodegradability of the process water.

**Fig. 1:**
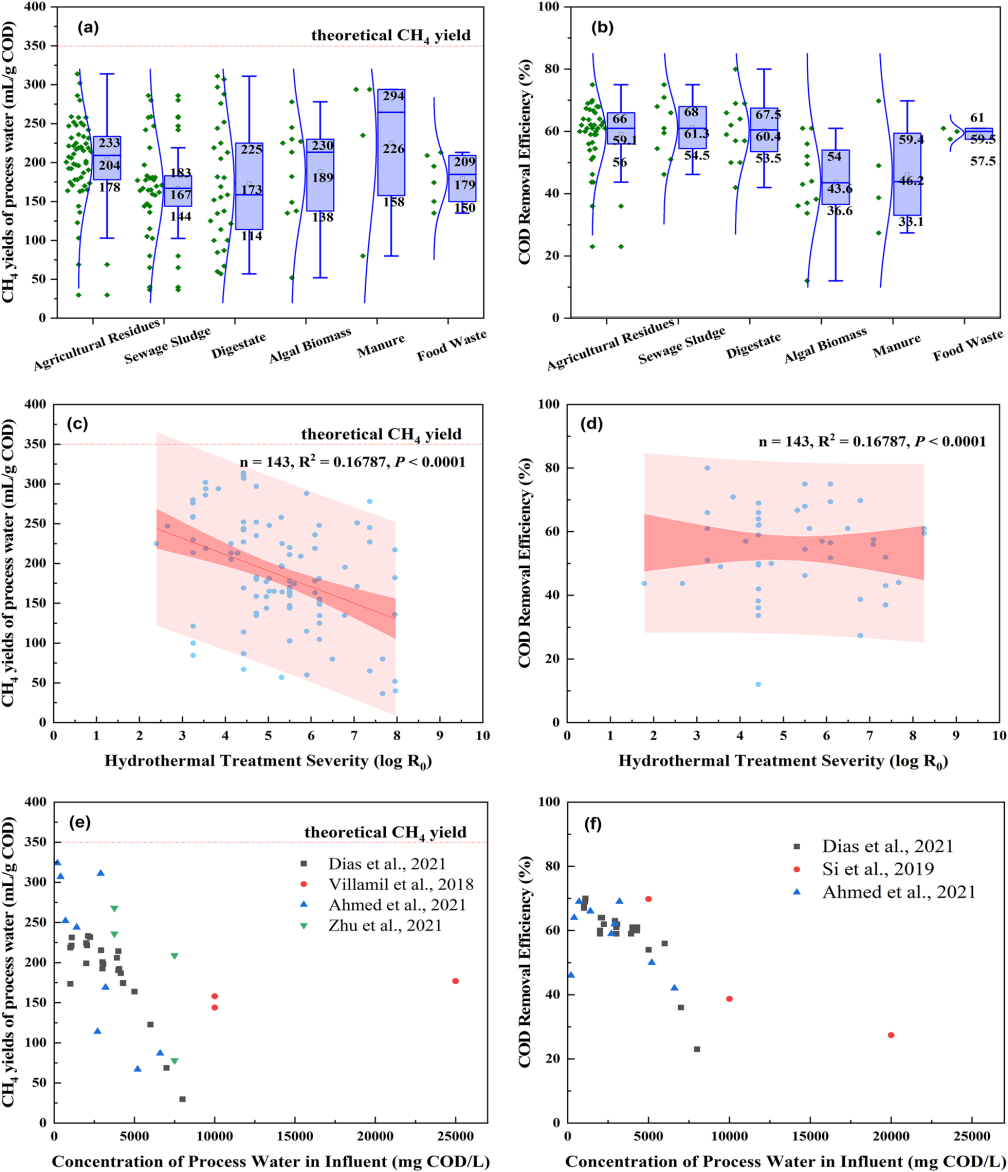
Effects of hydrothermal feedstocks (**a**, **b**), hydrothermal treatment severities (**c**, **d**), and concentration of process water (**e**, **f**) on methane yields and COD removal efficiency after AD treatment of process water. Data was collected from 30 studies and plotted with Origin 2023

CH_4_ yields from process water, based on the chemical oxygen demand (COD), range widely depending on the source–from 40 mL CH_4_/g COD to > 300 mL CH_4_/g COD (Fig. 1a). Based on the review of multiple studies (n = 30), process water from manure showed the highest average CH_4_ yields at 226 mL CH_4_/g COD, with a 50% probability distribution within 158–294 mL CH_4_/g COD (Marin-Batista et al. 2020; Si et al. 2019; Zhou et al. 2015). Process water from crop residues, food waste, and algae biomass resulted in similar CH_4_ yields. The CH_4_ yields from crop residues averaged 204 mL CH/g COD, with a 50% probability distribution within 178–233 mL CH_4_/g COD (Becker et al. 2014; Chen et al. 2017; Dias et al. 2021; Si et al. 2018; Wang et al. 2020; Wirth and Mumme 2013; Wirth et al. 2015; Xiang et al. 2021). The CH_4_ yields from food waste averaged 179 mL CH_4_/g COD, with a 50% probability distribution within 150–209 mL CH_4_/g COD (Erdogan et al. 2015; Mannarino et al. 2022). The CH_4_ yields from algal biomass averaged 189 ml CH_4_/g COD, with a 50% probability distribution within 138–230 mL CH_4_/g COD (Brown et al. 2020b; Li et al. 2019; Shanmugam et al. 2017; Tommaso et al. 2015; Yang et al. 2018; Yu et al. 2020; Zheng et al. 2017). Process water from sewage sludge and digestate resulted in lower CH_4_ yields: 167 mL CH_4_/g COD, with a 50% probability distribution within 144–183 mL CH_4_/g COD (Chen et al. 2019; De la Rubia et al. 2018; Gaur et al. 2020; Hao et al. 2020; Usman et al. 2019; Villamil et al. 2018; Wirth et al. 2015), and 173 mL CH_4_/g COD, with a 50% probability distribution within 114–225 mL CH_4_/g COD (Ahmed et al. 2021; Ipiales et al. 2021; Parmar and Ross 2019; Zhu et al. 2021). The average CH_4_ yields from process water differed depending on their source type; however, these differences may not be statistically significant, given the high variation observed between studies. The high variability within source types demonstrates the substantial impact of processing conditions on process water biodegradability.

COD removal efficiency data is less variable than methane yields (Fig. 1b). Most studies reported 40–70% COD removal during AD of process water derived from different feedstocks (Ahmed et al. 2021; Dias et al. 2021; Hao et al. 2020; Li et al. 2019; Si et al. 2018, 2019; Tommaso et al. 2015; Wirth and Mumme 2013; Wirth et al. 2015; Xiang et al. 2021; Yu et al. 2020). Process water from algal biomass showed an average CH_4_ removal efficiency lower than 50% (i.e., 43 ± 10%) (Fig. 1b), perhaps due to their higher protein content than other feedstocks (Chen et al. 2014; Lu et al. 2018). The higher protein content of feedstocks leads to high concentration of TAN and N-containing compounds such as pyrazines (Basar et al. 2023). High concentration of TAN could induce inhibition and pyrazines were reported to persist during AD treatment (Li et al. 2019; Wang et al. 2018).

### Effects of hydrothermal treatment severity

Reaction temperatures and residence time during hydrothermal processing have a more important effect on the properties of process water than feedstock composition (Posmanik et al. 2017). The impact of hydrothermal treatment conditions (i.e., temperatures and residence time) can be reported in terms of the severity factor (R_0_) (**Eq. **1), which facilitates the comparison of process water characteristics by partly normalizing differences in hydrothermal treatment conditions (Ahmad et al. 2018).

1 $$log\; {R}_{0}=log\left(t*{e}^{\frac{T-{T}_{ref}}{14.75}}\right)$$

where *t* is the residence time (min), *T* is the reaction temperature ( ℃), and *T*_*ref*_ is the reference temperature (100 ℃). Note: hydrothermal severity (*log R*_*0*_) increases with treatment temperature and residence time.

CH_4_ yields of process water decreased with increasing hydrothermal treatment severity from 4.28 (treatment conditions: 175 ℃ for 120 min) to 7.97 (treatment conditions: 320 ℃ for 120 min) (Fig. 1c), according to the data collected from previous studies (Ahmed et al. 2021; Brown et al. 2020a; Chen et al. 2019, 2017; De la Rubia et al. 2018; Dias et al. 2021; Erdogan et al. 2015; Gaur et al. 2020; Hao et al. 2020; Ipiales et al. 2021; Li et al. 2019; Mannarino et al. 2022; Marin-Batista et al. 2020; Parmar and Ross 2019; Shanmugam et al. 2017; Si et al. 2018, 2019; Tommaso et al. 2015; Usman et al. 2020; Villamil et al. 2018; Wang et al. 2020; Wirth and Mumme 2013; Wirth et al. 2015; Xiang et al. 2021; Yang et al. 2018; Yu et al. 2020; Zheng et al. 2017; Zhou et al. 2015; Zhu et al. 2021). A recent study also observed a negative correlation between reaction temperature and CH_4_ yields (Ma et al. 2024). The reaction temperature has a more pronounced effect on methane yields than residence time. For instance, Chen et al. (2019) reported that CH_4_ yields decreased from 286 mL CH_4_/g COD to 136 mL CH_4_/g COD when the severity increased from 3.54 (treatment conditions: 170 ℃ for 30 min) to 7.95 (treatment conditions: 320 ℃ for 30 min). Conversely, increasing the residence time from 60 to 120 min and 240 min while holding a constant temperature only marginally decreased CH_4_ yields from 183 mL CH_4_/g COD to 181 mL CH_4_/g COD and then to 164 mL CH_4_/g COD, respectively.

Hydrothermal treatment severity also has a minor influence on COD removal efficiency during AD of process water (Fig. 1d). Generally, COD removal efficiency correlates negatively with hydrothermal treatment severity. For instance, the COD removal was 59.5% for sewage sludge process water generated at a severity of 8.26 (320 ℃, 60 min) and 70.9% at 3.84 (170 ℃, 60 min) (Hao et al. 2020). In another study, the COD removal efficiency was only 36.9% for digestate solids process water generated at 14.31 (530 ℃, 45 min) and 56.9% at 8.43 (330 ℃, 45 min) (Hübner and Mumme 2015). Based on Fig. 1d, the mean value of the COD removal efficiency is around 58%, which is much lower than that from direct AD treatment of easily degraded substrates (> 85%) (Shanmugam et al. 2017).

### Effects of process water concentration

The elevated concentration of process water showed an adverse effect on CH_4_ yields (Fig. 1e). AD was stable at low concentration of process water (i.e., < 3.75 g COD/L), producing CH_4_ yields of 158–260 mL CH_4_/g COD (Ahmed et al. 2021; Dias et al. 2021; Villamil et al. 2018; Zhu et al. 2021). However, the methane yields were severely inhibited when the concentration of process water exceeded 5 g COD/L. For instance, CH_4_ yields decreased by 47.9% when the process water concentration increased from 5 g COD/L to 10 g COD/L (Si et al. 2019). Also, process water at 15 g COD/L reduced the CH_4_ yields by 73% compared to a of 3.5 g COD/L (Zhu et al. 2021).

COD removal efficiency responds similarly, ranging from 59% to 70% at process water concentrations lower than 3.75 g COD/L (Fig. 1f), and decreasing from 69.8% to 27.4% when the concentration increased from 5 g COD/L to 20 g COD/L (Si et al. 2019). The decreased COD removal efficiency indicated an inhibition of anaerobic conversion, supported by the reduced conversion of acid intermediates, specifically propionic acid, butyric acid, and valeric acid.

A prolonged lag phase in biogas production is a potential indicator of AD process inhibition (Li et al. 2023a). The slow degradation of complex substrates by unacclimated biomass could result in the lag phase. However, two studies investigating AD treatment of process water observed increasing lag phases with increasing doses of process water. The inclusion of 13.3% process water (*vol./vol.*) caused a lag phase of 8 days, while 26.7% (*vol./vol.*) caused nearly complete inhibition of the AD process (i.e., lag phase > 35 days) (Zhou et al. 2015). In another study, increasing the process water concentration from 5 g COD/L to 10 g COD/L and 20 g COD/L increased the lag phase from 5.9 days to 12.2 days and 36.3 days, respectively (Si et al. 2019).

## Toxicity of process water to anaerobic microbes

### Low pH toxicity

Process water can be highly acidic (with a pH between 3.5 and 5) and contains some organic acids, of which volatile fatty acids (VFAs) comprise the main fraction (Watson et al. 2020). Highly acidic process water can inhibit AD because the optimum pH range for single-stage AD is approximately from 6.8 to 7.2 (Cioabla et al. 2012; Usack et al. 2012). Although most organic acids in the process water, such as volatile fatty acids (VFAs), are readily biodegradable, they can inhibit the AD process under certain conditions. The onset and extent of VFA inhibition depend on multiple interacting factors, including, for example, the level of biomass acclimation, pH, temperature, substrate composition, cooccurrence of other inhibitors, and the type of VFAs present (Chen et al. 2008). The onset of moderate inhibition has been reported as low as 2.4 g/L (Wang et al. 2009), while severe inhibition can occur beyond a VFA concentration of 5 g/L. The AD process can also collapse entirely at VFA concentrations exceeding 10 g/L (Siegert and Banks 2005; Villamil et al. 2018).

**Fig. 2:**
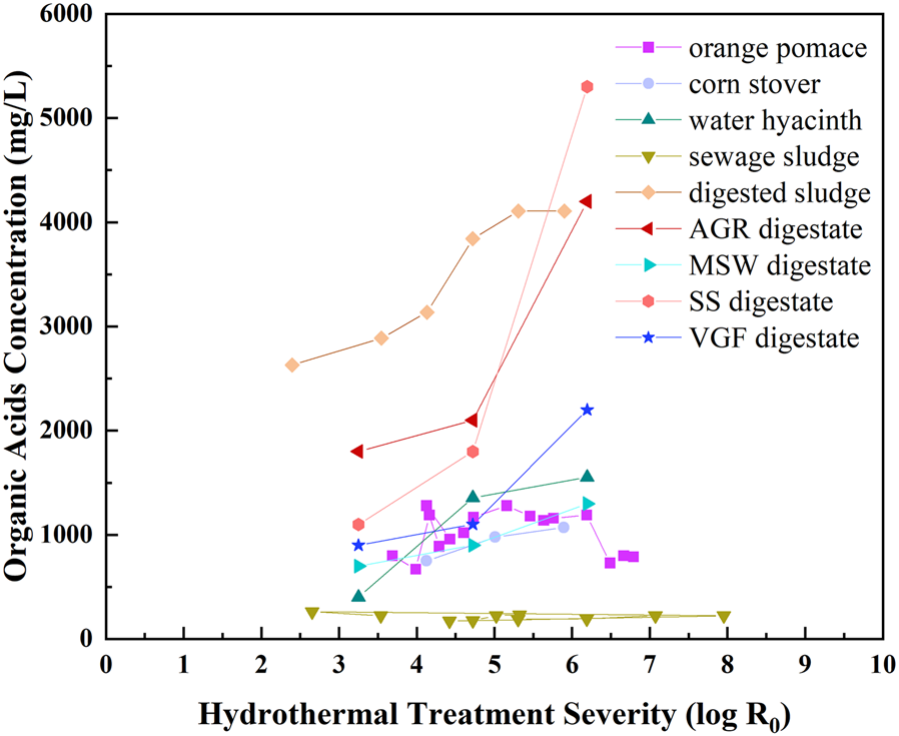
Correlation of hydrothermal treatment severity and organic acids concentration in process water derived from: orange pomace (Erdogan et al. 2015), corn stover (Wang et al. 2020), water hyacinth (Brown et al. 2020a), sewage sludge (Chen et al. 2019), digested sludge (Zhu et al. 2021), and agricultural residue (AGR), residual organic fraction of municipal solid waste (MSW), sewage sludge (SS), and organic household waste comprised of vegetable, garden, and fruit (VGF) (Parmar and Ross 2019). The data from the cited studies were collected and was plotted with Origin 2023

Hydrothermal treatment severities can be altered to reduce process water acidity when treating lignocellulose biomass or feedstocks rich in carbohydrates. Organic acids (excluding amino acids) are mainly formed from the hydrolysis of carbohydrates, hemicellulose, and cellulose (Chen et al. 2014). Higher concentrations of organic acids occur with increased reaction temperatures or residence time (Fig. 2). Therefore, limiting hydrothermal treatment severities to lower than five is helpful to avoid low pH toxicity. For instance, a residence time lower than 120 min is recommended at a process temperature of 200 ℃ during bio-crude oil production.

### High TAN toxicity

Inorganic total ammonia nitrogen (TAN) in process water, in the forms of ammonium (NH_4_^+^) and free ammonia (NH_3_), possibly leads to methane production and COD removal inhibition during AD treatment. The ratio of NH_3_/NH_4_^+^ (free ammonia speciation) increases with increasing pH and temperature (Usack and Angenent 2015). Moderate TAN concentrations (below toxic levels) help stabilize the AD process by buffering against pH drops that are caused by organic acid accumulation. Moreover, 0.2–1 g/L TAN concentrations benefit the AD process, providing nitrogen as an essential nutrient source (Filer et al. 2019).

However, TAN concentrations above 1.7 g/L must be avoided to prevent inhibition (Chen et al. 2008). Severe inhibition of methanogenesis occurred at TAN concentrations of 3.05–5.77 g/L (Sung and Liu 2003). In another study, the methanogenic population lost nearly 56.5% of its activity when exposed to TAN concentrations of 4.05–5.73 g/L (Chen et al. 2008). Unacclimated biomass can also be inhibited when free ammonia concentrations exceed ~ 80–150 mg NH_3_-N/L (Usack and Angenent 2015). Free ammonia is more inhibitory than ammonium because it readily permeates the cell membrane, disrupting the intracellular pH.

**Fig. 3:**
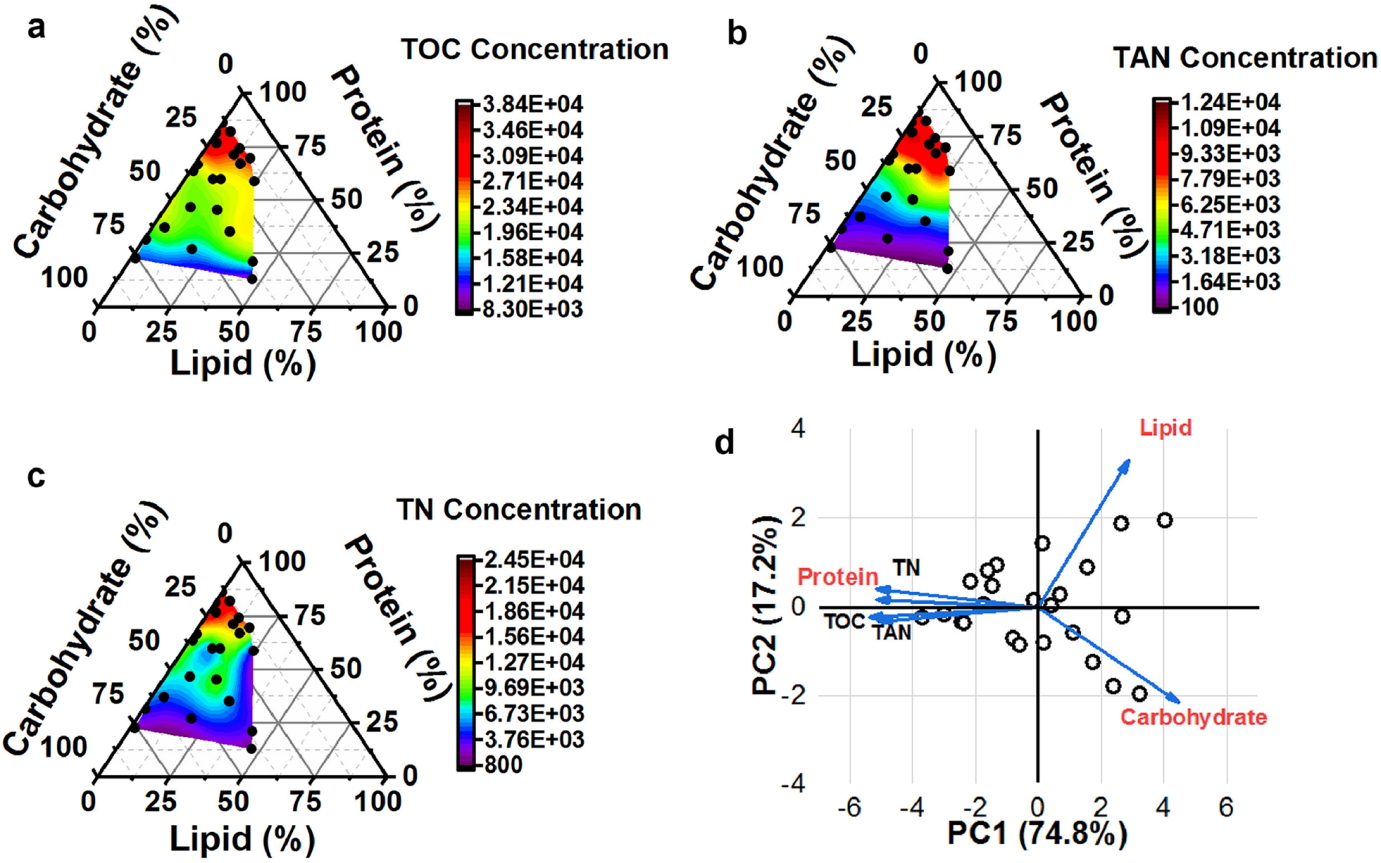
Characterization of hydrothermal process water generated from different feedstock compositions: total organic carbon (TOC) concentration (mg/L) (**a**), total ammonia nitrogen (TAN) concentration (mg/L) (**b**), total nitrogen (TN) concentration (mg/L) (**c**), and principal component analysis (PCA) (**d**). Reprinted from (Watson et al. 2020). Copyright © 2023, with permission from Elsevier

Watson et al. (2020) reviewed the total nitrogen (TN) stream in process water, consisting of TAN, organic nitrogen, and limited nitrate nitrogen content. Process water derived from high-protein substrates (i.e., manure and algae) contains high TN and TAN concentrations, making it more likely to cause TAN toxicity during AD (Fig. 3**)**. TAN forms primarily from protein hydrolysis and deamination reactions (Wang et al. 2018). These forms of toxicity are associated with reduced COD removal efficiency, as indicated in Fig. 1b for algae biomass and manure. In contrast, process water that was derived from lignocellulosic and carbohydrate-rich feedstocks is less susceptible to high TAN toxicity due to their limited protein content (Li et al. 2023b).

TN and TAN concentrations can also be changed by varying the hydrothermal conditions. The TN concentration of the process water increased with an increasing temperature and reached plateaus when the hydrothermal treatment temperature rose above 240 ℃ (Yu et al. 2011). Another study (Wang et al. 2018) also showed that TN concentration sharply increased from 180 ℃ to 220 ℃ but had no significant change after subsequent increases in temperature. However, the concentration of total organic nitrogen started to drop at temperatures above 220 ℃, while TAN concentration exhibited higher formation rates at temperatures above 220 ℃ (Wang et al. 2018). It was mainly due to the continuous conversion of protein to inorganic nitrogen in the process water. Therefore, high TAN toxicity can be curtailed by operating hydrothermal treatment at temperatures lower than 220 ℃ (Marin-Batista et al. 2020; Wilson and Novak 2009).

### Toxicity of organic compounds

In addition to organic acids and TAN, toxic organic compounds in the process water may limit energy recovery from AD due to inhibition (Shao et al. 2023). The toxic organic compounds in process water include aldehydes, phenols, alcohols, pyridine, and their derivatives (Li et al. 2019). Process water contains relatively high concentrations of furfural-type aldehydes, particularly 5-hydroxymethyl-furfural, which exert cytotoxic effects on bacteria and yeast by denaturing polynucleotides and damaging proteins within the cell (Wen et al. 2020). Furfurals comprise a furan ring with an aldehyde functional group. They usually form through dehydration and oxidation reactions with pentoses such as xylose and arabinose. Phenolic compounds are also prevalent in process water and induce toxic effects by disrupting the cellular membrane (Mills et al. 2009). Phenolic compounds are prevalent in the fruits, seeds, and vegetative tissues in plants. They comprise one or more hydroxyl groups attached to an aromatic benzene ring. N-heterocyclic compounds (pyridines, pyrroles) are another known inhibitor in process water, causing cellular disruption via non-specific enzymatic protein reactions (Shao et al. 2023). This class of compounds is characterized by heterocyclic rings where N constitutes the heteroatom.

The structure of toxic organic compounds can be an important factor in inhibiting methane production. Limited analysis showed that the toxicity of organic compounds in process water depends on the type and the number of substitutions. Pham et al. (2013) showed that the mammalian cell cytotoxicity of nitrogenous compounds (NOCs) with methyl groups (i.e., 3-dimethylamino-phenol, 2,2,6,6-tetramethyl-4-piperidone, and 2,6-dimethyl3-pyridinol) was higher than those without them. An increase in the number of hydroxyl groups on the aromatic compound was associated with a decrease in the compound’s toxicity to methanogens (archaea). The toxicity of various phenolic monomers decreased in the following order: pyrogallol < hydroquinone < resorcinol < phenol < benzene (Kayembe et al. 2013). However, synergistic effects due to multiple toxic compounds might be a more pertinent determinant of inhibition than individual compound concentrations. Still, more research is needed to distinguish the toxicity of individual organic compounds and their synergistic effects.

**Fig. 4:**
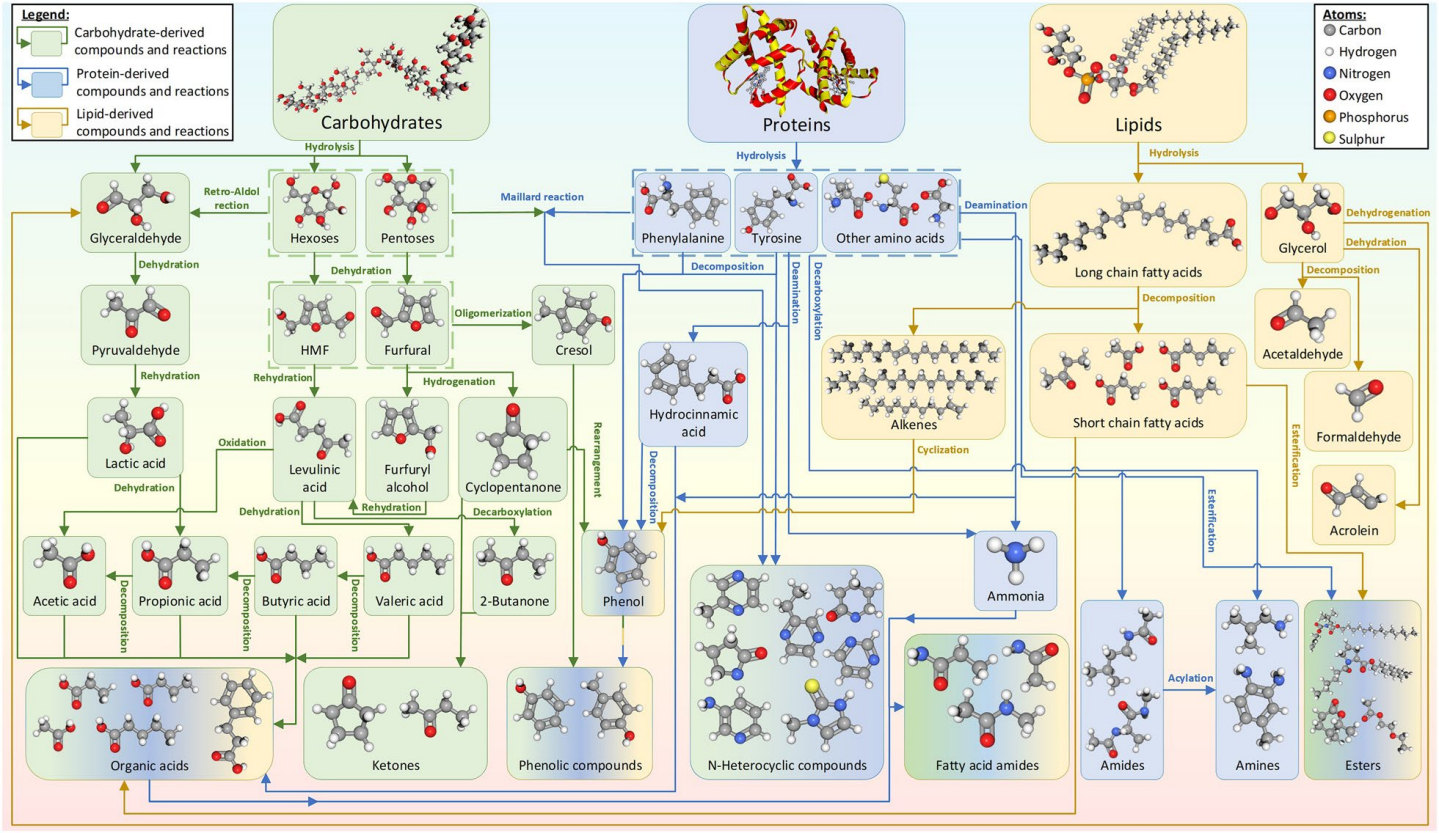
Hydrothermal formation pathways of compounds and groups detected in GC–MS analysis. Reprinted from (Basar et al. 2023). Copyright © 2023, with permission from Elsevier

Basar et al. (2023) summarized the formation pathways of different organic compounds during hydrothermal treatment (Fig. 4). Generally, protein-rich feedstocks lead to the formation of N-heterocyclic compounds. On the other hand, carbohydrate-rich feedstocks lead to the formation of furans, aldehydes, ketones, and phenolic compounds, especially at hydrothermal reaction temperatures exceeding 200 ℃. Lipid-rich feedstocks also contribute to the formation of toxic aldehydes (*e.g.,* formaldehyde, acrolein) and phenolic compounds.

Toxic compounds can interact synergistically in AD systems to cause more pronounced inhibitory effects than when present individually. Antagonistic interactions can also occur between inhibitory compounds (Chen et al. 2008). Process water typically contains multiple toxic compounds, and their relative proportions vary depending on the feedstock type and processing conditions. Anaerobic toxicity assays or biochemical methane potential tests with individual compounds or mixtures of compounds can be used to screen for potential toxicity. However, these assays are time-consuming, and their results weakly translate to continuously-fed AD systems (Posmanik et al. 2017). Also, analytical identification and quantification of specific compounds in the process water is costly and technically challenging. Instead, most studies use qualitative approaches to measure these compounds (Nguyen et al. 2023). However, as researchers continue to collect more quantitative data, there is a growing opportunity to develop predictive models that predict specific product formation during hydrothermal treatment as a function of initial feedstock composition and hydrothermal reaction conditions.

## Recalcitrant compounds limiting AD performance

Besides potential toxic compounds, the recalcitrance of some compounds in process water may also contribute to the low COD removal efficiency during AD treatment. A recalcitrant compound is one that degrades slowly or incompletely under standard operating conditions (Knapp and Bromley-Challoner 2003). The general approach to characterize the recalcitrance of process water during anaerobic degradation is to compare specific component concentrations in the AD influent and effluent. For instance, aromatic compounds are generally more recalcitrant than organic acids, and thus comprise a larger fraction of the residual compounds in AD effluent. However, ideally, multiple analytical methods should be used to gain more insight into which molecules are recalcitrant.

### Analytical methods

Robust analytical methods are key to tracking the transformation of compounds in the process water. Gas chromatography-mass spectrometry (GC–MS) has been the most widely used method for identifying various compounds in AD influents and effluents. However, precisely identifying and quantifying these compounds in the process water using GC–MS is challenging. Not all recalcitrant compounds can be detected by GC–MS (Chen et al. 2016; Si et al. 2019). Temperature limits of GC ovens prevent the identification of compounds with boiling temperatures higher than 400 ℃. Another challenge is the precision of the results. The National Institute of Standards and Technology (NIST) database provides the reference data for identifying peaks in the mass spectrum. Still, due to chromatograph complexity, the database cannot address peak overlaps, which often occur with process water and AD samples, especially when so many different compounds are present in a complex sample.

Other methods emerged to characterize process water in recent years. Electrospray ionization (ESI) coupled with Fourier-transform ion cyclotron resonance mass spectrometry (ESI FT-ICR MS) can determine basic molecular characteristics of complex organic compounds, such as aromaticity and double-bond equivalents. This technique, therefore, can reveal molecular-level transformations during water treatment processes (Hao et al. 2020). However, the absence of widely available standards limits the quantitative analysis by ESI FT-ICR MS (Hao et al. 2020; Yuan et al. 2017). Integration of liquid chromatography-organic carbon detection-organic nitrogen detection (LC-OCD-OND) is an alternative quantitative approach to track the transformation of different sub-groups, such as polysaccharides and proteins, and identify the organic compounds recalcitrant to AD (Chen et al. 2016).

### Recalcitrant compounds identified by GC–MS

GC–MS can identify certain heteroatom compounds. Heteroatoms are categorized into nitrogenous (containing N) and oxygenated (containing O but no N) groups. The nitrogenous group can be further divided into N-heterocyclic compounds (benzene heterocycle with nitrogen atoms), amides, and pyrroles. The oxygenated compounds mainly consist of carboxylic acids, alcohols, ketones, esters, aldehydes, phenols, and O-heterocyclic (benzene heterocycle with oxygen atoms).

GC–MS can help elucidate the transformation mechanisms of complex organic compounds during AD, but more robust quantification methods are still needed. For instance, the removal efficiency of recalcitrant compounds in process water is typically estimated by the change of peak area percentages in influent and effluent (Hao et al. 2020). Liu et al. (2023) noted that nitrogen-containing aromatics comprised most of the recalcitrant compounds that remained after AD treatment. This conclusion is corroborated by a previous study wherein pyrazines were identified as the most recalcitrant group (Table 2) (Li et al. 2019). Moreover, other groups of compounds have been found to persist at high levels, including various amines and cyclic ketones (Li et al. 2019), phenolic compounds (*e.g.,* phenol, 4-ethyl-phenol, 3-methyl-phenol, and 4-hydroxy-acetophenone) (Li et al. 2019; Si et al. 2018; Wirth and Mumme 2013), and benzoic acids (Si et al. 2018) (Table 2).

**Table 2:** Removal efficiency of major organic compounds in the process water identified by GC–MS

Groups	Compounds	Formula and structure	Removal efficiency (%)
Phenols	4-ethyl-2-methoxy-phenol		100^e^
	4-hydroxy-benzaldehyde		100^e^
	3,4-dimethoxy-phenol		100^e^, 100^g^
	2,6-dimethoxy-phenol		100^e^, 100^g^
	2-methoxy-phenol		100^g^
	Mequinol (4-methoxyphenol)		100
	Vanillin		100^e^, 100^g^
	Phenol		55.8^b^, 60^e^, 83^f^, 100^g^
	4-ethyl-phenol		54^e^, 100^g^
	4-hydroxy-acetophenone		59^e^
	3-methyl-phenol (3-cresol)		58^e^
Furans	furfural		100^e^, 100^g^
	5-hydroxymethyl-furfural (5HMF)		100^g^
	5-methyl-2-furancarboxaldehyde		100^e^
	2-furanmethanol		100^g^
Ketones	1-(2-furanyl)-ethanone		53.6^e^
	2-methyl-2-cyclopenten-1-one		100^e^
	2-acetyl-cyclohexanone		57^h^
	3,3,4,4-tetramethyl-cyclopentanone		45.5^a^
	2,4,6-trimethoxyacetophenone		30^d^
	2,3-dimethyl-2-cyclopenten-1-one		10.9^a^
Carboxylic acids	Butylated hydroxytoluene		100^b^
	Benzoic acid		85^e^
Alcohols	Neopentyl glycol		58.2^b^, 100^d^
	2-trimethylsilyl-ethanol		30%^c^
Pyridines	2-aminopyridine		100^d^
	3-hydroxy pyridine		56^e^
Pyrazines	2-ethyl-5-methyl-pyrazine		100^d^
	Ethyl-pyrazine		55.3^b^
	Pyrazine		53.2^b^
	Trimethyl-pyrazine		53.1^b^
	2,5-dimethyl-pyrazine		52.1^b^
	2-ethyl-6-methyl-pyrazine		49.2^b^
	Methyl-Pyrazine		45.8^b^
	3-ethyl-2,5-dimethyl-pyrazine		31.9^b^
Pyrimidines	5-methyl-7-amino-s-triazolo-pyrimidine		100^d^
Pyrroles	2-pyrrolidinone		90^f^
	N-[2-hydroxyethyl]-succinimide		100^b^
	N-[2-hydroxyethyl]-succinimide		100^b^
	Alpha-methyl-alpha propylsuccinimide		54.9^b^
Amino acids	amino acids		77.2^a^
Oxazine	2-amino-5,6-dihydro-4,4,6-trimethyl-4H-1,3-oxazine		100^b^
Amines	straight amides derivatives		87.6^a^
	N-(2-methylpropyl)-acetamide		54.1^b^
	N-(3-methyl butyl) acetamide		43^b^
	N-acetyl-2-ethylbutan-1-amine		35.2^b^

References: a. swine manure process water (Yang et al. 2018); b. algae process water (Li et al. 2019); c. sewage sludge process water (Hao et al. 2020); d. cow manure process water (Marin-Batista et al. 2020); e. cornstalk process water (Si et al. 2018); f. corn silage process water (Wirth and Mumme 2013); g. corn stover process water (Wang et al. 2020); h. digested sludge process water (Zhu et al. 2021); formulas and structures (Kim et al*.*, 2023)

During hydrothermal processing, N and O-heterocyclic compounds are mainly produced from the Maillard reaction between amino acids from the hydrolysis of proteins and reducing sugars from the hydrolysis of carbohydrates (Gai et al. 2015). N and O-heterocyclic compounds are water soluble, toxic, and resistant to anaerobic biodegradation. Maillard reactions intensify at 180°C; therefore, reaction temperatures beyond 180°C and residence times longer than 15 min tend to cause higher N and O-heterocyclic compound concentrations (Chen et al. 2019).

Besides N and O-heterocyclic compounds, amine and cyclic oxygenate derivatives are minimally degraded during AD. For instance, previous studies detected various aromatics and N-structures (Mannarino et al. 2022) from process water in AD effluents. Benzene and its derivatives, such as fluorobiphenyl (FBP), have been considered the most toxic and persistent hydrocarbon petroleum constituents (Leng and Zhou 2018). The aromaticity of benzene confers high structural stability, making it particularly resistant to oxidization and degradation (Si et al. 2018).

### Recalcitrant compounds identified by multiple methods

Based on LC − OCD-OND analysis of process water, biopolymers including high molecular weight polysaccharides and high molecular weight protein-like substances showed limited biodegradation after AD (Hao et al. 2020). Although compounds with low molecular weights are generally more biodegradable, some neutral low-molecular-weight compounds (including alcohols, aldehydes, ketones and mono-oligosaccharides) with more hydrophobic and aromatic structures can be recalcitrant (Li et al. 2016), as exhibited by the lower COD removal efficiency (54.3%) from 320 °C process water compared to 170 °C process water (74.3%), where they were less prevalent (Hao et al. 2020).

## Approaches to improve AD performance

### Mitigate toxicity

#### Low pH and high TAN toxicity

Acid stress on AD can be mitigated by pH adjustment with basic or buffering chemicals such as NaOH, Ca(OH)_2_, Mg(OH)_2_, KH_2_PO_4_, NaCO_3_, and NaHCO_3_ (Yang et al. 2015). Similarly, alkali stress–while less prevalent an issue–can be mitigated by supplementing acids such as H_2_SO_4_ or HCl. The main drawback of using chemicals and buffers to control pH is cost. Also, the addition of acids causes CO_2_ dissolution, which may result in excessive foaming in the AD system, causing instability (Usack and Angenent 2015). An ideal strategy to remediate process water toxicity would not involve using pure chemicals.

Based on the analysis in "Low pH toxicity" and "High TAN toxicity" sections, organic acids and TAN concentrations in the process water can be controlled by lowering hydrothermal severities. Combining protein-rich into carbohydrate-rich feedstocks makes it possible to adjust the final pH of the process water after hydrothermal treatment (Gai et al. 2015). For example, Adedeji et al. (2023) successfully amended process water for AD treatment by adjusting the hydrothermal feedstock mixture. Also, dilution before AD treatment can be an effective *post-hoc* intervention for low-pH and high-TAN process waters. Dilution reduces the concentration of problematic species (*e.g.,* organic acids, TAN, and other toxic compounds) while simultaneously reducing the organic strength of the process water. However, dilution increases the volumetric throughput during AD, creating more effluent for disposal.

A simple but somewhat expensive approach for TAN removal is struvite precipitation. When the molar ratio of NH_4_^+^: Mg^2+^: PO_4_^3−^ ions are at 1:1:1 under alkaline conditions, ammonium will be removed in the form of magnesium ammonium phosphate hexahydrate (MgNH_4_PO_4_⋅6H_2_O, struvite) (Shanmugam et al. 2017). For example, Wang et al. (2021) achieved 76.5–80.8% ammonium removal by adding MgCl_2_⋅6H_2_O and KH_2_PO_4_ to process water. Moreover, they observed that pH affected TAN removal efficiency (tested pH values: 7.0, 7.5, 8.0). The highest removal rate occurred at pH 7.5. When sufficient PO_4_^3−^ is present in process water alongside TAN, only Mg^2+^ is needed for struvite precipitation (Fettig et al. 2019). Overall, pre-treating process water with struvite precipitation is an effective strategy to prevent TAN inhibition during AD. Shanmugam et al. (2017) measured 35% higher methane yields during AD from process water treated with struvite precipitation compared to untreated process water. The drawback of struvite precipitation is the cost of Mg^2+^ and PO_4_^3−^ supplementation. Removing 1 kg NH_4_^+^, requires 1.35 kg MgCl_2_⋅6H_2_O and 5.27 kg KH_2_PO_4_.

Using zeolite, TAN can be removed from process water through ion exchange and adsorption. Zeolite has been widely used for ammonium-rich wastewater due to the presence of Na^+^, Ca^2+^, and Mg^2+^ in its crystalline structure (Zheng et al. 2017). The biogas yield from process water increased considerably from 59.1 to 245.3 mL/g COD to 78.0–331.3 mL/g COD following zeolite pretreatment (Ruirui et al. 2017). However, the effectiveness of zeolite depends on the ammonium concentration. Zeolite could not effectively remove TAN at concentrations lower than 4.92 g/L (Zheng et al. 2017).

Simultaneous nitrification–denitrification could be another possibility for TAN removal with oxygen-limiting intermittent aeration (i.e., microaeration) (Feng et al. 2018). No one has reported the application of simultaneous nitrification–denitrification on hydrothermal treatment process water during AD treatment. Only Macedo et al. (2023) investigated the possibility of recycling process water derived from hydrothermal sludge treatment to the municipal wastewater treatment stages. They studied the effect of process water on nitrification and denitrification. Ammonium oxidizing bacteria were inhibited by process water at COD concentrations corresponding to 89 mg O_2_/L and more severely affected by the increasing concentration of process water. Process water showed lower inhibition to the nitrite-oxidizing bacteria, which was not affected by process water up to COD concentrations of 134–223 mg O_2_/L. Notably, nitrifying activity recovered after a short-term inhibition of the nitrification due to a shock-load of process water. Furthermore, process water acted as a carbon source to facilitate denitrification.

### *Toxicity of organic compounds*

Adding adsorbents mitigates the toxicity of process water by removing toxic organic compounds before AD. Popular absorbents include granule activated carbon (GAC) and powdered activated carbon (PAC). It has been reported that GAC pretreatment can effectively remove more than 50% of phenolic compounds. Methane production rate and the COD removal efficiency generally increase with increased GAC loading (Wang et al. 2021). Adding PAC in the digester also improved AD performance, reducing the lag phase from 35 to 23 days (Zhou et al. 2015). However, these adsorption-based approaches do not solve the problem of toxic compound treatment because the compounds persist in the adsorbate. Still, they render process water more benign to AD, allowing AD to treat the remaining organic compounds. Finally, adsorption-based approaches are non-specific, which means a fraction of the biodegradable compounds will also be entrapped.

Considering the COD losses due to adsorption pretreatment and the high prices of GAC and PAC, in-situ co-treatment during AD may be more advantageous. Hydrocar, the solid phase separated from hydrothermal treatment processes, could be added to digesters along with the process water (Periyavaram et al. 2023; Yang et al. 2023). Hydrochar acts more like a buffer that could temporally store organics and slowly release them to the microbes, contributing to a higher methane yield and higher conversion rate (Zheng et al. 2017). Adsorption on hydrochar concentrates toxic and recalcitrant compounds within the adsorption zone, reducing biomass exposure in the bulk liquid zone. However, in one study, supplementing hydrochar and process water seemed to induce severe inhibition during AD (Zhu et al. 2021), possibly due to pH-related or competitive adsorption effects.

The general concept of detoxification is to reduce the concentration of toxic organic compounds in the process water. Adsorption is the most widely used detoxification approach. However, since adsorption is non-specific, removing toxic compounds also reduces the COD of process water. A more effective strategy to improve AD performance is transforming recalcitrant or toxic organic compounds into more degradable and less toxic forms.

### Degrade recalcitrant compounds

#### Current approach

Wet air oxidation (WAO) has proven to be an effective process for treating wastewater with high organic matter content, allowing the total or partial degradation of toxic or recalcitrant compounds for biological treatment (de Mora et al. 2024). During WAO, organic compounds are oxidized into primarily acetic acid and formic acid by applying high temperatures (175–350 ℃) and high O_2_/air pressures (20–90 bar), generating a stream of readily degradable compounds for subsequent AD treatment. For example, WAO treatment at 50 bar air pressure and 250 ℃ significantly reduced phenolic, ketone, aromatic, and olefin compounds in process water (Kilgore et al. 2023). Moreover, with the addition of catalysts, it was possible to eliminate these compounds almost completely. However, WAO was not as effective in removing pyrazines (de Mora et al. 2024). Also, while WAO is an effective strategy to degrade a broad spectrum of process water contaminants simultaneously, it has its drawbacks. One drawback is its high energy requirements for pressurization and heating (Silva Thomsen et al. 2022). Another drawback is that WAO completely oxidizes a fraction of the organic compounds to CO_2_, precluding their recovery as biogas (Kilgore et al. 2023). Finally, the scale-up of WAO is technically challenging and prohibitively expensive due to its high-pressure requirements. Ried et al. (2007) also demonstrated the treatment of refractory organics in industrial wastewater by integrating ozone systems in biological treatment schemes. Ozone addition (2.1 mg O_3_/mL HTL process water) increased the maximum methane yield by 37.5% but led to a more extended lag phase of 21.3 days compared to 12.6 days with untreated HTL process water (Yang et al. 2018). The improvement in methane production was attributed to the conversion of recalcitrant organics into more biodegradable compounds. For instance, after ozonation, phenols were fully converted to organic acids, and around 21.7% of N-heterocyclic compounds were oxidized (Yang et al. 2017). At the same time, however, ozonation was shown in another study to generate new toxic compounds not present within the initial wastewater (de Souza et al. 2010), suggesting ozone would severely disturb the microbiome at higher doses.

#### Perspectives of new approaches

Because the degradation of aromatic compounds is difficult in the absence of oxygen during AD (Boll et al. 2002), creating a microaerobic environment that is less detrimental than ozone may be promising. The combination of anoxic-aerobic processes was reported to enhance the biodegradation of pyridine with an anaerobic baffled reactor (ABR) and a moving-bed biofilm reactor (MBBR) (Shen et al. 2015). The NH_4_^+^ released from pyridine biodegradation in the ABR was nitrified completely into NO_3_^−^ in the MBBR, which was then recirculated back to the ABR, serving as the electron acceptor for pyridine biodegradation. Jiang et al. (2018) developed an electricity-assisted anaerobic system to enhance the biodegradation of pyridine by stimulating the compact biofilm and the microaerobic environment at the anode. However, these methods still need further investigation and development.

During microaeration, water electrolysis and various oxygen-dosing techniques can be applied to AD systems to promote the growth of facultative bacteria, whose potent enzymes may enhance the degradation of toxic and recalcitrant compounds (Magdalena et al. 2022). Gavazza et al. (2015) applied 2.5 and 3.0 V electric potentials to produce oxygen *in-situ* via water electrolysis. They noticed that oxygen promoted the aerobic degradation of aromatic amines. Another study (Kim et al. 2016) achieved microaeration by saturating the feed medium headspace with air. Oxygen or air dosing is the most-used microaeration method in the AD process. Oxygen or air can be supplied to the influent (do Nascimento et al. 2021), gas phase (Ortiz-Ardila et al. 2021), or liquid phase (Jenicek et al. 2014) of the digesters. Exploring more efficient methods for oxygen dosing is crucial because of the low solubility of oxygen in water and the poor gas–liquid transfer. Also, these dosing methods cannot quantify or control oxygen transfer precisely.

## Concluding remarks

Coupling hydrothermal treatment and AD treatment of process water represents a promising strategy to improve overall resource recovery from wet feedstocks. The recovery of organics from process water as biogas enhances the environmental benefits and economic viability of hydrothermal treatment. However, the complexity of processing water makes its biodegradability unpredictable, rendering AD performance unreliable. Moreover, high concentrations of organic acids, TAN, and toxic compounds in process water destabilize the AD process. Finally, the recalcitrant compounds in process water resist biological degradation and persist in the AD effluent, sometimes necessitating subsequent treatment using other technologies.

**Fig. 5:**
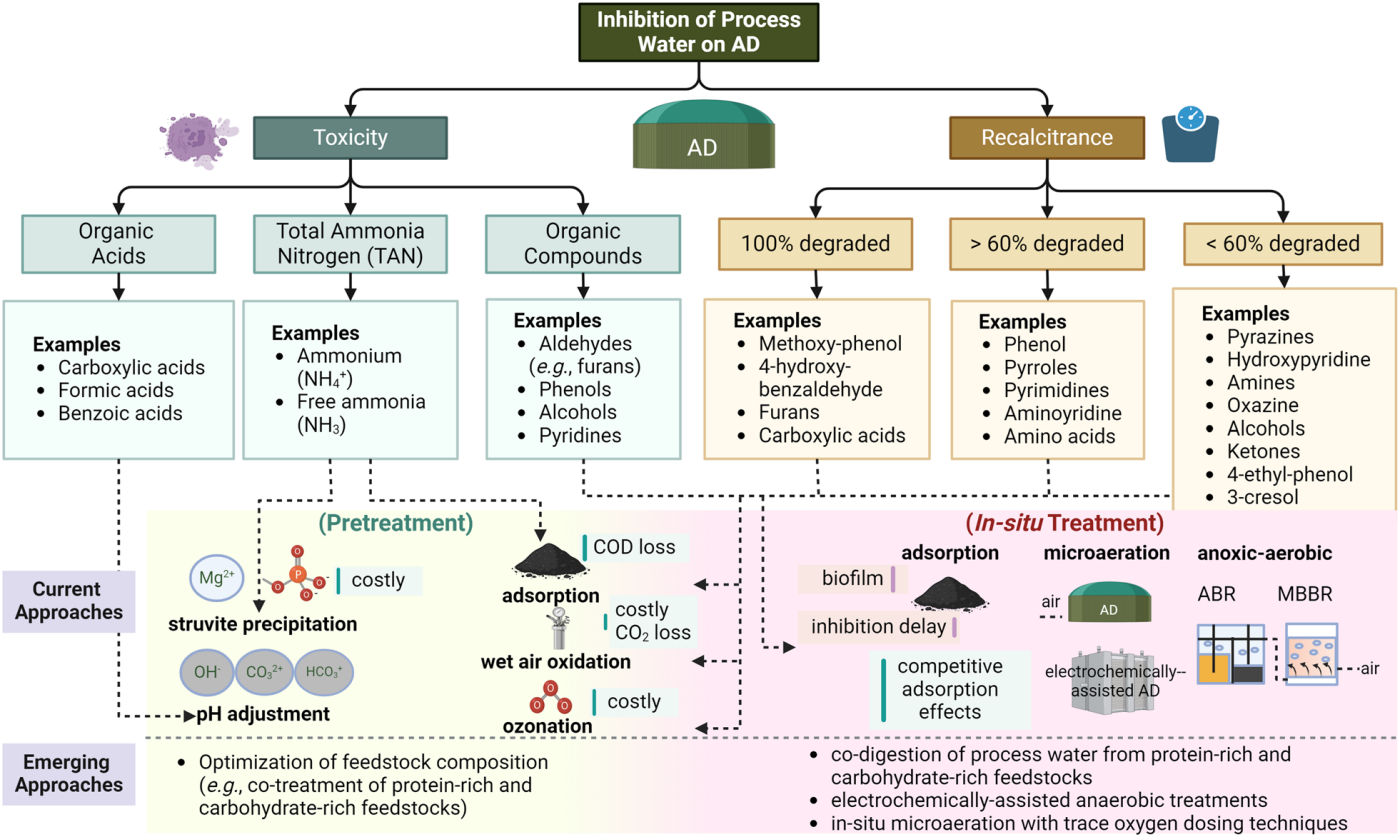
Overview of AD inhibitors in the process water and detoxification approaches. Created with BioRender.com

Thus, multiple challenges must be addressed and specific questions answered to facilitate the valorization of process water using AD treatment (Fig. 5). Developing robust strategies to quantify recalcitrant components is urgently needed, as current characterization methods make distinguishing the cause of diminished AD performance difficult. The effects of individual inhibitors and the co-effects on AD performance are also worth further study. Another important endeavor is to develop a mechanistic understanding of process water degradation in anaerobic systems, which would help optimize the AD treatment process.

Although some studies have reported the benefits of adsorption for removing TAN and some toxic compounds, new strategies should be pursued to mitigate inhibition without a collateral loss of the labile organics in the process water. Advanced techniques such as WAO, ozonation and microaeration can help oxidize recalcitrant compounds. However, further efforts are needed to refine these techniques to avoid unintended consequences caused by their misapplication.

## Data Availability

All data generated or analyzed during this study are included in this published article.

## Abbreviations

AD: Anaerobic digestion
COD: Chemical oxygen demand
VFAs: Volatile fatty acids
TAN: Total ammonia nitrogen
TN: Total nitrogen
AGR: Agricultural residue
SS: Sewage sludge
VGF: Organic household waste (comprised of vegetable, garden and fruits)
GC–MS: Gas chromatography-mass spectrometry
ESI FT-ICR MS: Electrospray ionization (ESI) coupled with Fourier-transform ion cyclotron resonance mass spectrometry
LC-OCD-OND: Liquid chromatography-organic carbon detection-organic nitrogen detection
GAC: Granule activated carbon
PAC: Powdered-activated carbon
WAO: Wet air oxidation
ABR: Anaerobic baffled reactor
MBBR: Moving-bed biofilm reactor
